# A Structured Approach to Online Learning Design in Dental Education

**DOI:** 10.15694/mep.2020.000246.1

**Published:** 2020-10-30

**Authors:** Cassandra Lewis

**Affiliations:** 1 Queen Mary University London

**Keywords:** Online learning design tool, e-learning, healthcare education, dental education, transition to clinical practice, evidence-based teaching

## Abstract

This article was migrated. The article was marked as recommended.

The transition to online learning is an accepted and anticipated change across all higher education institutions. These changes have become even more relevant to healthcare education given the challenges posed by COVID19. This paper describes the application of the Cambridge Education Group Pedagogic Framework (2018) to online dental education, specifically the conversion of a face-to-face ‘Transition to Clinical Practice’ module in paediatric dentistry. The framework has a foundation in medical education and holds great value for clinical academics across all healthcare disciplines in the design and implementation of online teaching. This affords educators much needed structure and assistance in meeting the needs of our students in this era of online learning. The advantages and disadvantages are explored, illustrated by student feedback, following a pilot implementation at a Dental School in the United Kingdom.

## Introduction

The Cambridge Education Group Pedagogic Framework ( CEG, 2018) is based on the Course Design Sprint (CoDesignS) Framework (2020); a pedagogic method for designing and developing online learning activities, founded by the University of Liverpool and Imperial College London ( Toro-Troconis *et al.*, 2016). The original 2016 version of this framework was aimed at practical, workplace-based learning within medical education. It mapped practical tasks to frameworks and standards for higher education in the United Kingdom (UK), specifically those by the Academy of Medical Educators (2014) and the General Medical Council (2016). Furthermore, credibility and usefulness to academics is found in the framework aligning teaching practices with academic promotion and progression domains, for example those recognised in the ‘UK Professional Standards Framework’ (UKPSF) by Advance HE (2011).

The framework is adaptable, and the same approach can be adopted across different disciplines, institutions and countries as per their respective educational standards. Therefore, its application to online dental education seemed both relevant and timely. Mapping to the expected learning outcomes and standards for dental education was indeed straight-forward, and educators are directed to those produced by the General Dental Council ( GDC, 2015a; 2015b) and Health Education England ( HEE, 2018).

This paper describes the application of the CEG framework to online dental education, specifically the conversion of a face-to-face ‘Transition to Clinical Practice’ module in paediatric dentistry, illustrated by student feedback, following a pilot at a Dental School in the United Kingdom.

## Implementation of the Framework

Traditionally, a face-to-face ‘Transition to Clinical Practice’ module in paediatric dentistry is provided for 3rd year dental undergraduates, centred on clinical observation, peer-learning, reflection and case-based learning in a situated outreach environment to enable students to comfortably and confidently progress to clinical practice. The CEG Framework was used to convert this teaching to a format suitable for online delivery, and in mapping to relevant educational standards frameworks ( Table 1). This is an important stage in dental training; indeed, students report high levels of stress and anxiety during the transition to clinical practice, which can negatively affect their learning or ability to learn ( Godefrooij, Diemers and Scherpbier, 2010; Alzahem *et al.*, 2011; Elani *et al.*, 2014; Botelho, Gao and Bhuyan, 2018; Serrano *et al.*, 2018; Atherley *et al.*, 2019). Therefore, teaching in this period must be robust, relevant, and supportive.

The six CEG framework phases were used to convert this teaching to a format suitable for online delivery following COVID19 lockdown. The implementation of these phases will be exemplified in their application to this module, concluding with qualitative student feedback in highlighting its merits and challenges.

### Phase I: The Scope of the learning Intervention.

This stage involves exploration of the expectations of the module. What are the aims and objectives of the teaching? How is this best delivered online? What are the numbers of students and availability of staff? Is it a single learning episode, a week or a whole module? What are the digital competencies of your learners and staff; will all involved have the same access to the internet? In this context, a 12-week e-learning module was required, focused on the transition to clinical practice in paediatric dentistry for 3 ^rd^ year undergraduate students. Neither students nor staff had prior experience with online learning. A key requirement was for all online activities to be recorded and/or accessible to students unable to attend for various reasons (different time zones, limited internet access etc).

### Phase II: Learning Outcomes.

This stage involves determination of learning outcomes, and relates to basic pedagogic principles in classifying learning outcomes based on Bloom’s taxonomy of learning ( Anderson and Krathwol, 2001). Based on the pre-existing face-to-face teaching, six key topics relevant to paediatric dentistry were identified and assigned learning outcomes, mapped to those set by the GDC (2015a). Discussions with colleagues were invaluable for determining relevance and appropriateness in blueprinting to the existing curriculum, and any changes related to COVID19.

6 Topics:

1. Non-pharmacological behaviour management
2. Consent, History & Examination
3. Caries Risk Assessment & Prevention
4. Radiography, Special Tests & Classification of Carious Lesions
5. Choice of Restorative Options for Paediatric Dentistry
6. Treatment Planning in Paediatric Dentistry

The CEG Framework (2018) and Course Design Sprint (CoDesignS) Framework (2020) provide guidance in the design, selection and alignment of learning outcomes with learning activities which is in keeping with Bigg’s principles of constructive alignment ( Biggs and Tang, 2011). Colour-coded cards relate Bloom’s taxonomy verbs with appropriate learning activities for high-order cognitive skills (blue) e.g. discuss, interpret, analyse, apply, compare; or low-order cognitive skills (green) e.g. list, define, recall, differentiate.

### Phase III: Activity Descriptors.

Following design of learning outcomes, appropriate learning activities should be considered within the scope of digital technology. Due consideration must be given to the context, content and delivery; will teaching involve the whole year group, small groups or individuals? What material can be taught asynchronously using self-directed learning or a virtual learning environment (VLE), and what requires synchronous ‘live’ webinars? Will students require access to external tools e.g. online voting software or YouTube? Frequently a blended approach is most appropriate, and is preferred by students ( Morton *et al.*, 2016; Varthis and Anderson, 2018; Vanka, Vanka and Wali, 2020).

Recent literature suggests that participation in collaborative online activities has a positive effect on student performance ( Toro-Troconis and Aleksiev, 2018; Toro-Troconis, Alexander and Frutos-Perez, 2019). It is recommended that high-order cognitive skills are best addressed with collaborative approaches which support reflection and discussion, while low-order cognitive skills which are focused on factual and procedural knowledge, are more suited to self-directed learning modalities ( Toro-Troconis, 2015; Vanka, Vanka and Wali, 2020).

### Phase IV: Learning Descriptors.

In Phase IV the CEG Framework supports educators in aligning learning activities to learning types (descriptors) identified by Laurillard (2012) - specifically *enquiry, acquisition, discussion, practice* and *production.* For each learning descriptor, the cards suggest a suitable activity. For example; learning by *enquiry* through encouraging questioning and knowledge-seeking using existing learning resources; learning by *acquisition* through activities centred on listening, reading or watching; learning by *discussion* through collaborative activities in groups (with or without the tutor), and learning by *practice* and *production* through application of knowledge in the creation of something concrete e.g. a video, report or presentation. Many activities can be made available offline e.g. reading material, while others involve internet access to a VLE e.g. quizzes or discussion forums, or video software e.g. Blackboard collaborate or MS Teams.

To facilitate the blended online teaching of a cohort of 70-80 students over 12 weeks, the dental school VLE was used to host a combination of online asynchronous and synchronous activities in support of all five learning descriptors. Table 1 illustrates the process of aligning learning outcomes with teaching activities, mapping to relevant educational standards and frameworks within the UK context.

**Table 1.** The mapping of three example learning outcomes to learning activity, learning descriptor, HEE and UKPSF Framework Domains

Learning outcome *(Bloom’s Taxonomy level in italics)*	Learning Activity	Learning Descriptor (Laurillard, 2012)	HEE Framework Domains (2018)	UKPSFDomains (2011)
***List*** four factors which contribute to a paediatric patient’s caries risk *Remember*	Essential Reading of clinical guidelines (asynchronous)	Acquisition	3 and 5	A1,A2 K1,K2
***Analyse*** and ***interpret*** patient risk factors to carry out caries risk assessment of paediatric dental patients *Understand/Analyse*	Online quiz (asynchronous) and Webinar (synchronous)	Practice Discussion	3 and 5	A1-A4 K1-K4
***Reflect*** on how this knowledge can be applied to clinical practice *Evaluate*	Online Reflection and Discussion Forum (asynchronous)	Enquiry Discussion	3, 5 and 6	A1-A4 K1-K4
***Apply*** an evidence-based approach to treatment planning a paediatric dental patient *Apply/Create*	Webinar for case-based discussion (synchronous)	Enquiry Practice Discussion Production	3, 5 and 6	A1-A4 K1-K4
**Abbreviations:** HEE (Health Education England), UKPSF (UK Professional Standards Framework)				

### Phase V: Selection of Learning Activities.

This stage involves the organisation and scheduling of teaching activities. The CEG Framework suggests a 5-step process; *introduction* (linking current teaching with previous and future practice), *guided practice* (videos, podcasts, reading), *challenge activity* (quiz, discussion forum), *reflection* and *webinar.* This provides structured learning from knowledge acquisition to application, with reflection completing the learning cycle ( Hill, 2007) through the development of self-reflective attitudes and skills for life-long learning and self-monitoring ( Boud and Falchikov, 2007). Given the nature of all online teaching, the format needs to be clearly communicated, consistent and organised. Therefore, the six Topics were spread over 12 weeks (giving 2 weeks per Topic) with each following the same structure - asynchronous online ‘Essential Reading’, ‘Quiz’ and ‘Reflection & Discussion Forum’, followed by a synchronous ‘Webinar’ for case-based learning.

Teaching was organised so that progression through the Topics permitted focus on increasingly higher cognitive levels and competencies. Specifically, Miller’s Pyramid of competence levels of ‘Knows’ and ‘Knows How’, to ‘Shows How’ in the final Topic on treatment planning ( Miller, 1990). Quiz questions allowed formative assessment throughout the module, utilising written short-answer questions and multiple-choice questions which are considered appropriate in this setting ( Shumway and Harden, 2003; Albino *et al.*, 2009; Williams *et al.*, 2016).

The effectiveness of case-based learning is well-recognised in the literature ( Thistlethwaite *et al.*, 2012), particularly in linking theory to practice ( Kaufman, 2003; Spencer, 2003) and in supporting students in the transition to clinical work ( Williams, 2005). Encouraging student-centred learning ( Sambell, McDowell and Montgomery, 2013) through engagement in making personal judgements and defending clinical decisions promotes deep learning through scaffolding and constructivist learning principles ( Postma and White, 2016). Group discussion of findings and opinions on patient management aids in the development of reflective thinking and broader conceptual understanding of topics through collaborative learning and communities of practice ( Lave and Wenger, 1991).

### Phase VI: Analysis of Learning.

The CEG Framework (2018) advises educators to calculate the time spent on each type of learning, recommending a 70:20:10 balance of time based on work by Lombardo and Eichinger (1996) - 70% of learning activities focusing on learning through *experience* (enquiry, practice, and production), 20% on learning through *others* (discussion and collaboration) and only 10% on acquisition of knowledge through *didactic methods.* This is a challenge early on in all healthcare education where there is a need for theoretical grounding in the subject matter. Therefore, it is reasonable that this split may be weighted towards acquisition earlier on in the module, with more application of knowledge, discussion and collaboration towards the end. This approach was adopted in this module, with a 55:15:30 initial split in Topic 1, with acquisition reducing to 10% by Topic 6.

## Feedback: Students and Staff

Student feedback was collated through online questionnaire midway through the module. Overall it was extremely positive for the ‘Essential Reading’, ‘Quiz’ and ‘Webinar’ components, with students rating 88%, 94% and 80% for these methods respectively in terms of being ‘extremely’ or ‘very useful’ for their learning. Qualitative written feedback corroborated this, with students praising the design and structure of the module, commenting that they felt the ‘Quiz’ and ‘Webinars’ helped “test” and “apply” knowledge through putting it “into context”. Students also referenced the benefit of focusing reading on evidence-based guidelines for future revision, and in preparation prior to the webinars, with 96% reporting that 2 weeks was enough time to undertake each Topic. Furthermore, 96% of students felt this teaching had improved their confidence in seeing patients on clinic.

The asynchronous ‘Reflection & Discussion Forum’ was less well received, with only 35% finding this useful (indeed 24% felt it was not useful at all). This seemed largely due to students feeling there was not enough tutor feedback, expressing a preference for this to be discussed verbally. Whilst daily staff involvement in the discussion forum could improve this, it would be laborious and inefficient. Therefore, in response to student feedback, reflection was incorporated as a key component alongside case-based learning in subsequent webinars.

Staff also reported positive feedback in relation to flexibility and time-management. Indeed, the module will remain as a permanent fixture in the curriculum next academic year.

## Discussion: Adaptations and Future Use

While time-consuming to set-up, an online module can be easily maintained and updated for subsequent teaching. The initial challenge to the novice e-learning developer is familiarity with the VLE, the use of the quiz and questionnaire tools and navigating online video software. However, once mastered these afford flexibility for application elsewhere, and are essential skills for academics given the global movement in favour of online learning. Coordination of staff for departmental discussion and evaluation is also relatively straight-forward with the use of online video conferencing.

The CEG Framework provides a robust and user-friendly method for organising and delivering high-quality online teaching, which is applicable across disciplines. Whilst it is no substitute for practical, ‘hands-on’ experience, it allows for a structured approach in designing online learning both theoretically and practically, whilst supporting academic progression through alignment with educational promotion domains.

Although not included in the CEG framework, evaluation of the effectiveness of teaching is vital for both quality assurance and enhancement, and is an essential requirement of Higher Educational Institutions. In this module, the midway feedback allowed for feed-forward in real time ( Nicol and Macfarlane‐Dick, 2006) in support of student involvement in their own learning. Furthermore, staff could benefit through self- and/or peer-assessment by evaluating their recorded webinars, for example, referencing standards from the Five-Stage Model by Salmon (2011) for online interaction; student engagement, collaboration and scaffolded learning. This would further support quality assurance, and recognition of training needs, in this new era of online learning ( Fuhrmann and Weissburg, 1978; Wanner and Palmer, 2018).

## Take Home Messages

• The transition to online learning requires rapid adjustments to our teaching, learning and assessment processes.
• There is a need for simple and practical methods for the efficient design of robust online learning and teaching activities in healthcare education.
• Educators in health professions must improve their knowledge and skills in e-learning technologies and practices.
• The Cambridge Education Group Pedagogic Framework (2018) affords educators a robust and user-friendly method for organising and delivering high-quality online teaching.
• Student feedback is positive following a pilot online blended learning approach at a Dental School in the United Kingdom.

## Notes On Contributors

Cassandra graduated from Warwick University in 2008 with a BSc (hons) in Biological Sciences before completing her dental degree at Queen Mary University in 2012. Following graduation she worked within the Community Dental Services for several years before becoming a Clinical Lecturer in Paediatric Dentistry at QMUL. She is currently clinical lead for the BDS3 paediatric curriculum and lead for dental electives, whilst providing dental care under sedation for the NHS Trust following completion of the Post Graduate Diploma in Conscious Sedation in 2017. Her clinical interests include paediatric dentistry, dental anxiety and dental education. Cassandra is a Fellow of the Higher Education Academy and is currently undertaking the Masters in Medical Education at Dundee University.

ORCID ID: https://orcid.org/0000-0002-5568-0520
