# Supplementary material for: A Structured Approach to Online Learning Design in Dental Education
Source: MedEdPublish (2016). 2020 Oct 30;9:246. [Version 1] doi: 10.15694/mep.2020.000246.1 (PMC10697546; doi:10.15694/mep.2020.000246.1)
Supplement: Peer review history

# Reviewer report: Reviewer response for version 1

**DOI:** 10.21956/mep.20176.r31195

**Published:** 2020-11-16

**Authors:** Alexandra Webb

**Affiliations:** 1 Australian National University

This review has been migrated. The reviewer awarded 3 stars out of 5 Congratulations on this interesting article describing a practical experience of transitioning from face-to-face to online delivery using a structured framework.The article would benefit from a clearer explanation of the impetus to move to online delivery. The descriptions of each Phase of the Framework together with how these were applied in the context of this module are very helpful to link the theory and practice. If possible, it might be helpful to the reader to visually distinguish these different parts of the text. There does not appear to be an example of an application for Phase III: Activity Descriptors.‘Blended learning’ and ‘case-based learning’ are described but how they are relevant to and used in the context of this educational intervention is not outlined – it would be helpful to elaborate using examples from this pilot intervention.It would be interesting to include how the online module integrated with clinical/practical experiences within the program.The Framework and examples provided are a useful guide for anyone navigating the creation of online learning activities. The 70:20:10 to balance time is very helpful and sharing the challenges and solutions in applying this in your educational environment were insightful.

Reviewer Expertise:

NA

No decision status is available

# Reviewer report: Reviewer response for version 1

**DOI:** 10.21956/mep.20176.r31196

**Published:** 2020-11-05

**Authors:** Emma Kennedy

This review has been migrated. The reviewer awarded 4 stars out of 5 A concise and clear case study of online learning in the important domain of clinical practice. As the author notes, this is an area where teaching quality is vital, as students’ anxiety makes them vulnerable to disengagement. This is also an area where some might say it is harder to teach online, making the article an even more important intervention - in the Covid-19 context, yes, but also beyond this, given the likely persistence of online learning. The article uses a useful model (the Cambridge Education Group Model) and evidences a good sense of the theoretical background to this, providing a useful outline of how the CEG model works and its application in the context of dental clinical practice. Although the article focuses on dentistry, it would be a useful starting point for anyone in medical or health professions education as well, as it links the two vital domains of digital and clinical education. Lewis provides a clear and accessible summary of how long-held theories in education - Bloom’s Taxonomy and Miller’s Pyramid, for example - can be linked to twenty-first century learning design. The practical details from the evaluation are a useful insight into the strengths and weaknesses of this approach, and the article highlights a key issue around discussion forums: although many learning design theories, including this one, advocate asynchronous discussion forums, students consistently prefer synchronous discussion. I might have liked to see a little more detail around which topics worked better and why - for example, were there any topics or themes in clinical practice that did not seem to work as well for online learning? - but this is a minor point, and the article’s concision is also a strength. More study is certainly needed, both longer-term and in different domains - hopefully this article will provide a useful springboard to that study. I would recommend this article to anyone interested in digital learning design in dental, medical or health professions education.

Reviewer Expertise:

NA

No decision status is available

# Reviewer report: Reviewer response for version 1

**DOI:** 10.21956/mep.20176.r31197

**Published:** 2020-11-02

**Authors:** Tarun Sen Gupta

**Affiliations:** 1 James Cook University

This review has been migrated. The reviewer awarded 4 stars out of 5 This paper is a practical and useful addition to the peri-Covid medical education literature. While situated in dental education - which is in itself significant given the impact the pandemic has had on dental services and education - the issues and approaches outlined are generic, and likely to be applicable across many disciplines and jurisdictions.The paper presents some interesting approaches to curriculum design, notably structured around the six CEG framework phases, which provides an organizational approach that may be helpful in other settings. The 5-step process outlined may also be useful to others, similarly the 70:20:10 model (Lombardo and Eichenger). Table 1 (mapping of three example learning outcomes to learning activity, learning descriptor, HEE and UKPSF Framework Domains) will be particularly useful to readers exploring approaches to conversion to online content in a systematic manner. Being able to clearly articulate how these elements map to each other is often a challenge.In common with other peri-Covid literature, longer term evaluations going beyond student satisfaction are needed. I look forward to seeing production of such material in the future, along with recommendations for what we might retain in a PC (post-Covid) world.

Reviewer Expertise:

NA

No decision status is available
